# Clinical history correlates with lateral atlantoaxial (C1-2) joint edema. A pilot study

**DOI:** 10.1016/j.inpm.2024.100434

**Published:** 2024-09-04

**Authors:** Joshua Levin, Matthew Kaufman, Gerald Yeung

**Affiliations:** a Department of Orthopaedic Surgery, Stanford University, 450 Broadway St, Pavilion C, 4th Floor, MC 6342, Redwood City, CA, 94063, USA; b Department of Neurosurgery, Stanford University, 450 Broadway St, Pavilion C, 4th Floor, MC 6342, Redwood City, CA, 94063, USA

**Keywords:** Atlantoaxial, Joint, C1-2, History, Edema, Arthrosis

## Abstract

**Background:**

Clinical evaluation in the determination of the etiology of axial spine pain is limited.

**Objective:**

To determine if a set of three features of the clinical history are indicative of C1-2 joint edema on MRI.

**Methods:**

All patients from one physician's practice who had axial cervical spine pain from 2021 to 2023 were evaluated. Cases were defined as those with all 3 of the ASL criteria, defined as Age >65, Superior cervical/suboccipital pain, and exacerbation of pain primarily by Lateral cervical spine rotation. Age-matched controls had axial cervical spine pain without meeting the ASL criteria. Edema around the atlantoaxial joint and/or odontoid was evaluated by STIR MR sequences.

**Results:**

The ASL criteria had a sensitivity of 82 % [95 % CI: 64–100 %], specificity of 79 %, [95 % CI: 63–95 %], positive predictive value of 74 % [95 % CI: 54–94 %], and negative predictive value of 86 % [95 % CI: 72–100 %] in diagnosing C1-2 joint edema.

**Conclusion:**

A positive ASL criteria is sensitive and specific in the diagnosis of C1-2 joint edema, which may have clinical implications.

## 1 Introduction

Obtaining a good medical history has been referred to as “the greatest art in medicine” [1].

A patient's clinical history has been shown to be the most important aspect of the diagnostic evaluation in multiple areas including cardiovascular, neurologic, respiratory, urinary, and miscellaneous conditions [2]. Yet in the evaluation of axial spine pain, clinical evaluation has been shown to have limited diagnostic utility. While a history of episodic low back pain is indicative of discogenic pain [3], the clinical evaluation of other etiologies of low back pain, including lumbar facet joint pain [4], sacroiliac joint pain [5], and vertebrogenic low back pain [6], is less useful in obtaining an accurate diagnosis.

Diagnosing the etiology of neck pain is particularly challenging, partly due to overlapping referral patterns at different spinal levels [7]. However, the atlantoaxial (C1-2) joint is structurally and functionally unique. This joint is responsible for 80 % of cervical spine rotation [8], and degenerative changes of this joint are associated with suboccipital neck pain [9]. The prevalence of lateral atlantoaxial joint pain in neck pain patients is estimated to be 9 % [10], but as high as 50–62 % in patients with occipital headaches who are suspected of having C1-2 joint pain based on negative previous third occipital nerve blocks [11], or based on clinical features alone [9]. Given the unique nature of this joint, there may be specific clinical features alone that can accurately diagnose it as the etiology of neck pain with reasonably high diagnostic confidence.

MRI with fat suppression by STIR (short tau inversion recovery) sequences allows for the easy detection of edema. This may have clinical utility in the diagnostic evaluation of spinal pain [12,13]. Specific to the atlantoaxial joint, C1-2 joint inflammatory synovitis has been shown on contrast-enhanced MRI in rheumatoid arthritis patients with higher disease activity [14]. However unfortunately, there is no gold standard diagnostic or clinical test for C1-2 joint pain. Therefore, in a pilot study, we sought to determine if a combination of 3 clinical features, referred to as the ASL criteria – Age >65 years, Superior cervical/suboccipital neck pain, and exacerbation of pain with Lateral cervical spine rotation - was a reliable indicator of edema of the lateral atlantoaxial joint and/or odontoid, thereby potentially providing clinically relevant information. If so, a larger, prospective study from multiple providers would be pursued.

## 2 Methods

This study was approved by our academic medical center's institutional review board (IRB #71249) for a single site retrospective observational case control study, and the study was conducted according to the Declaration of Helsinki. A retrospective chart review was performed evaluating consecutive patients from one academic spine physiatrist's outpatient clinics between 2021 and 2023. The clinic notes from all new patients with axial cervical spine complaints were evaluated to find patients who met all 3 of the ASL criteria: Age >65, superior cervical/suboccipital pain, and exacerbation of pain exclusively or predominantly with cervical spine lateral rotation to either or both sides. Twenty-six consecutive age-matched controls with axial (non-radicular) cervical spine complaints who did not meet these criteria were included in the control group. Patients were included in the analysis if they had an MRI with fat suppression sequences available to review in our electronic medical record that was obtained either within six months of the reported onset of pain or their presentation for evaluation. A spine physiatrist with 15 years' experience in reviewing spine imaging (JL) evaluated the images for the presence or absence of lateral atlantoaxial and/or odontoid edema. A secondary analysis was performed on patients who had x-rays with sufficient views of the lateral atlantoaxial joints (preferably open-mouth views.) The reviewer was blinded to whether the images came from patients in the case or the control group.

## 3 Statistical analysis

Patients were dichotomized into cases (age >65, superior cervical/suboccipital pain, and exacerbation of pain exclusively or predominantly with cervical spine lateral rotation to either or both sides) and controls (age >65 and axial cervical spine pain not fitting the above criteria.) Each patient's imaging was evaluated for the presence of edema around the lateral atlantoaxial joint and/or the odontoid. A secondary analysis was performed evaluating for the presence of C1-2 joint arthrosis on x-ray on patients who had x-ray images sufficiently evaluating the C1-2 joints (ideally open-mouth views). For each analysis, sensitivity, specificity, positive likelihood ratio, and prevalence odds were calculated to determine the diagnostic confidence odds of the clinical presentation, and 95 % confidence intervals were calculated. Positive and negative predictive values were also calculated.

## 4 Results

**Fig. 1:**
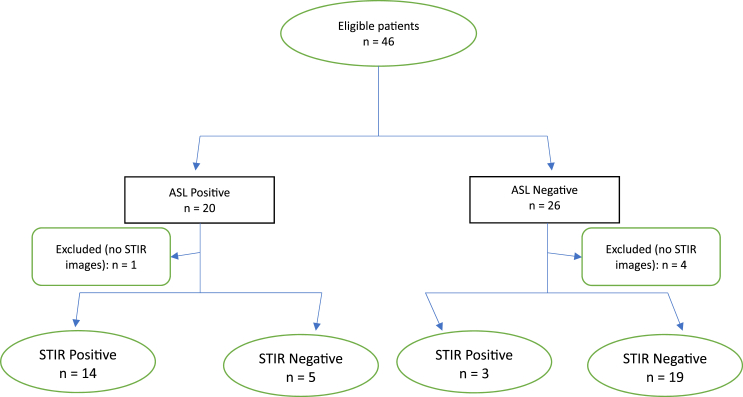
Flow diagram. ASL = Age, Superior cervical/suboccipital pain, exacerbated by Lateral rotation; STIR = short tau inversion recovery (on MRI).

A total of 20 cases and 26 controls were reviewed. One case and four controls were excluded due to the absence of STIR images, leaving a total of 19 cases and 22 controls who were included in the primary analysis of the MRI findings (Fig. 1). Demographic information including age and sex is shown in Table 1.

**Table 1:** Demographic information.

	Cases (Positive ASL criteria)	Controls (Negative ASL criteria)
Age (mean)	73.4 years	73.1 years
Female	11/19 (58 %)	9/22 (41 %)

ASL = Age, Superior cervical/suboccipital, Lateral rotation.

For the primary analysis, in determining if the ASL criteria correlated with edema of the C1-2 joint on MRI, 14 cases were positive while 5 were negative, and 3 controls were positive while 19 were negative (Table 2). The sensitivity of the ASL criteria was 82 % [95 % CI: 64–100 %], the specificity was 79 % [95 % CI: 63–95 %], and the positive likelihood ratio was 3.9. The positive predictive value (PPV) and negative predictive value (NPV) based on a prevalence of 41 % were 74 % [95 % CI: 54–94 %] and 86 % [95 % CI: 72–100 %], respectively. The diagnostic confidence odds were 2.7, with a diagnostic confidence of 0.73. See Fig. 2a, Fig. 2b, Fig. 3a, Fig. 3b, Fig. 3c for examples of C1-2 joints with arthrosis on X-ray and edema on MRI. Of note, 11/14 of the cases had C1-2 joint edema exclusively on the side of the patient's symptoms (or for patients with bilateral symptoms, it was on the more painful side), 2/14 had edema bilaterally, and one had edema only in the dens. None of the 14 cases had edema exclusively on the asymptomatic/less symptomatic side. Of the three control patients with edema, two had edema only in the dens, and one had edema on the asymptomatic side.

**Table 2:** Contingency table for Positive ASL criteria and C1-2 joint MR STIR images.

	STIR positive joint	STIR negative joint
Cases (Positive ASL criteria)	14	5
Controls (Negative ASL criteria)	3	19

ASL = Age, Superior cervical/suboccipital, Lateral rotation; MR = magnetic resonance.

STIR = short tau inversion recovery.

**Fig. 2a:**
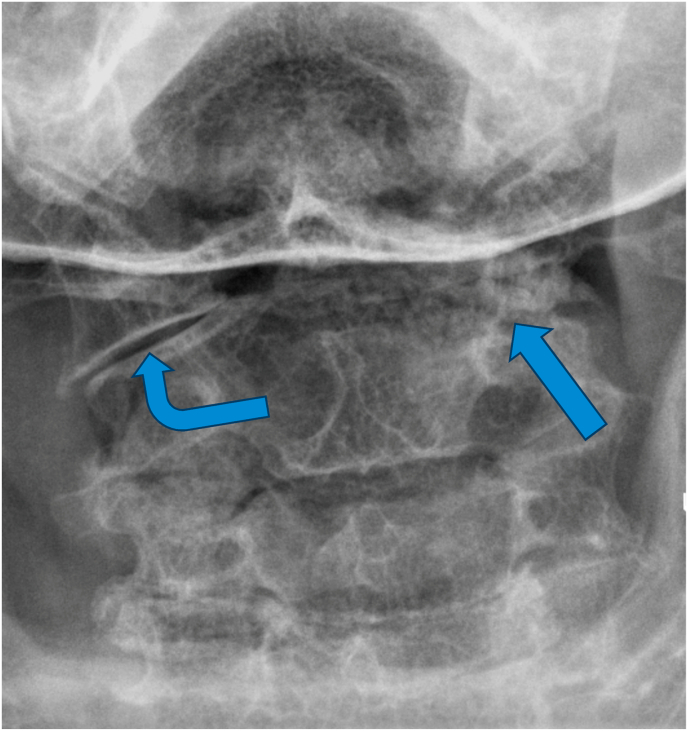
X-ray from a patient with left suboccipital pain demonstrating severe left C1-2 joint arthrosis (straight arrow) with a well-preserved joint on the right side (curved arrow).

**Fig. 2b:**
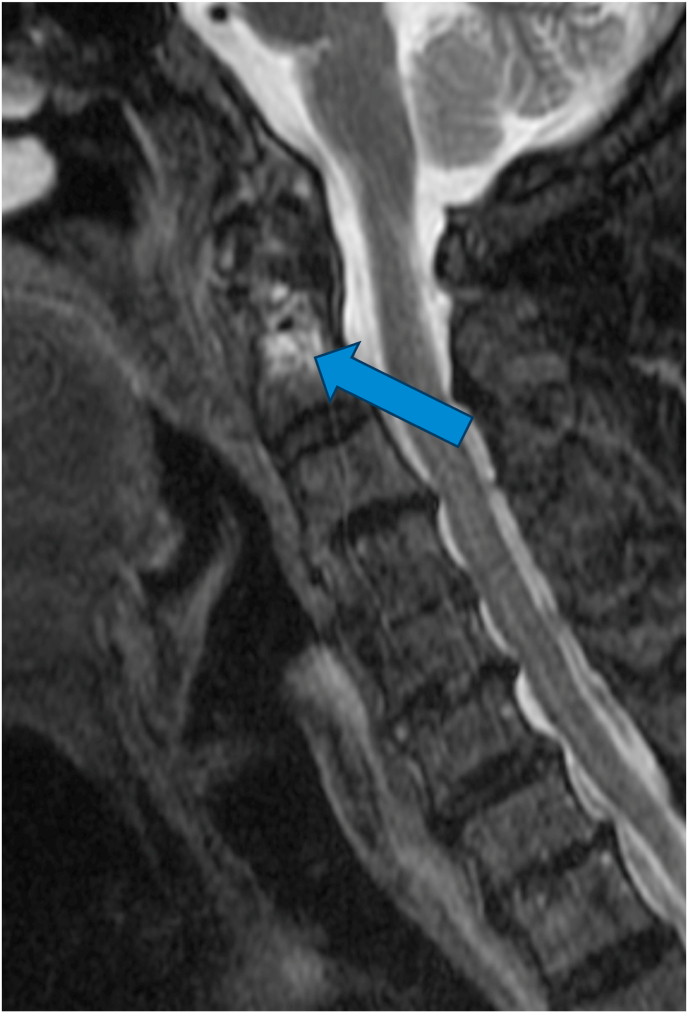
MRI showing edema in the dens (straight arrow).

**Fig. 3a:**
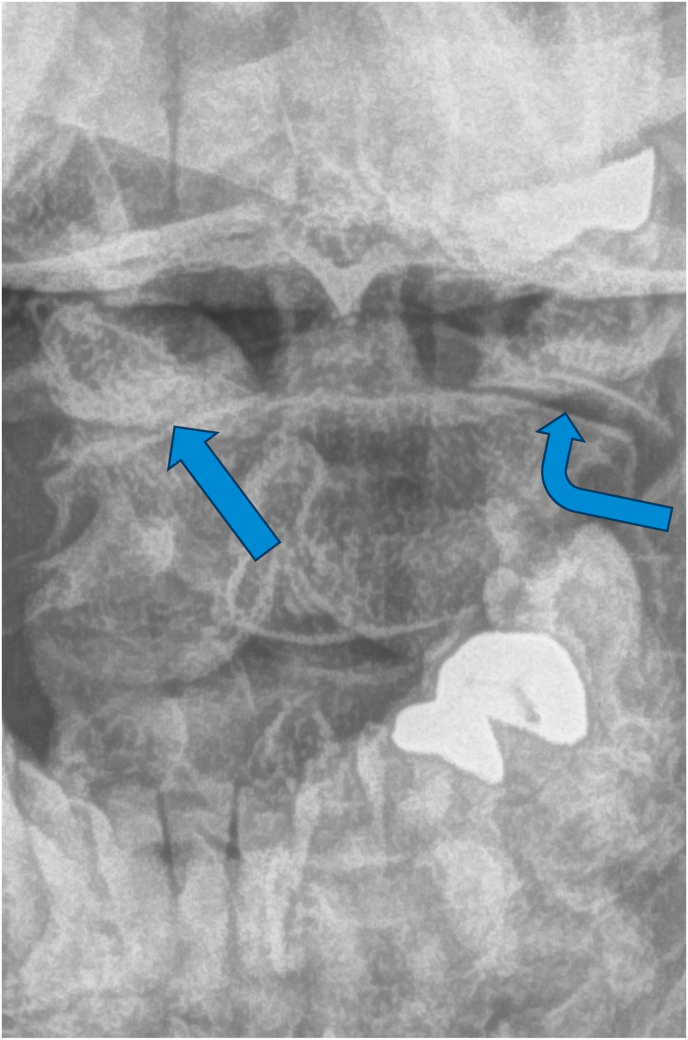
X-ray from a patient with right suboccipital pain demonstrating severe arthrosis of the right C1-2 joint (straight arrow) with a well-preserved joint on the left side (curved arrow).

**Fig. 3b:**
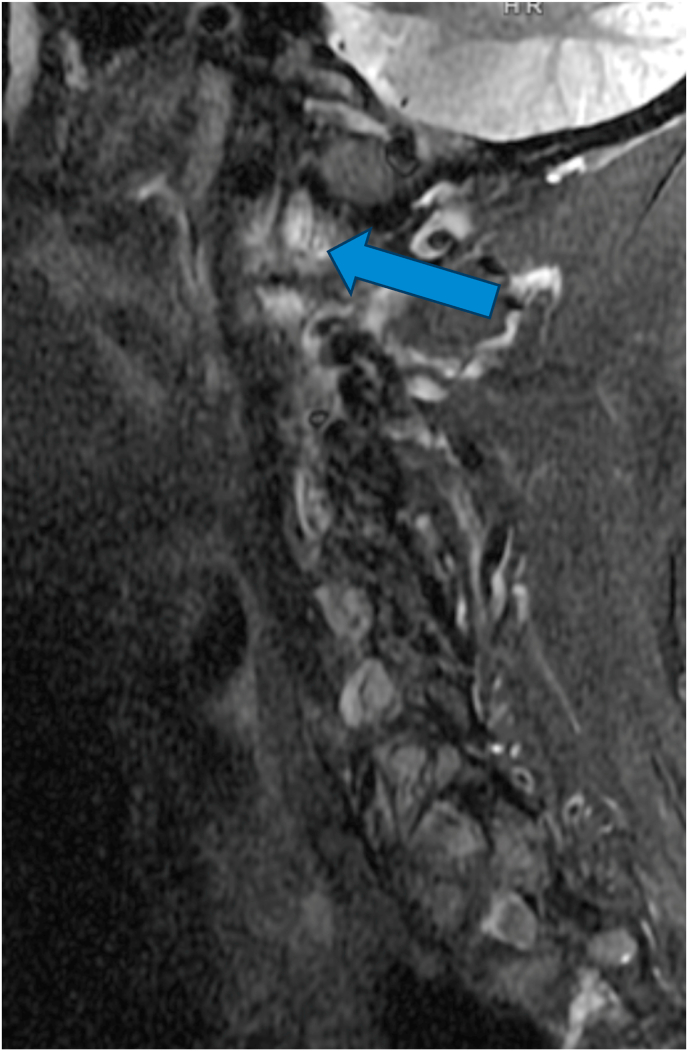
MRI showing edema in the right C1-2 joint (straight arrow).

**Fig. 3c:**
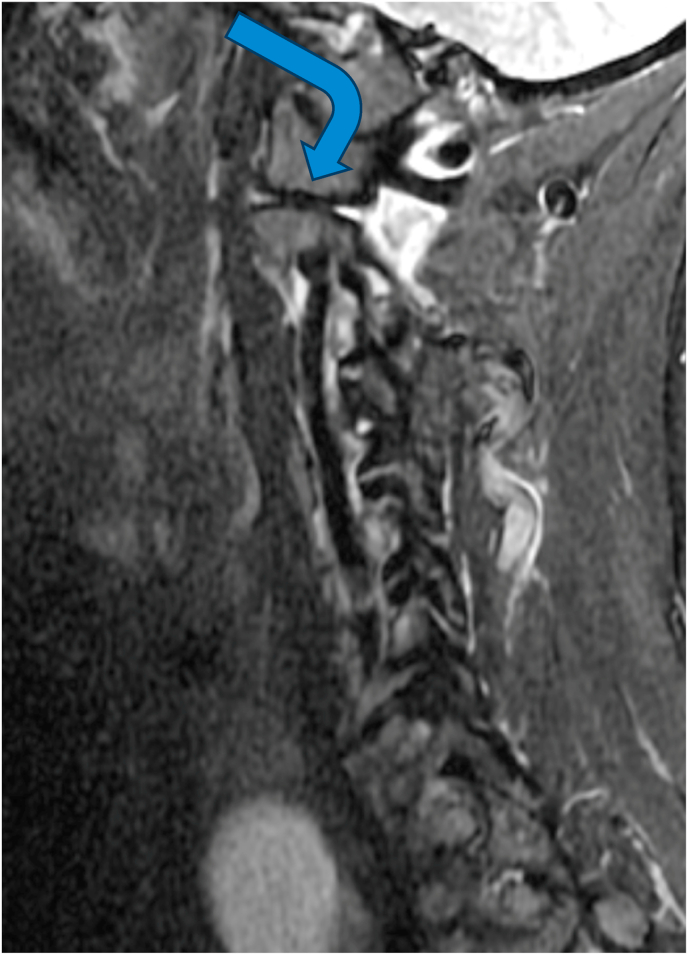
MRI showing no abnormalities in the left C1-2 joint (curved arrow).

**Fig. 4:**
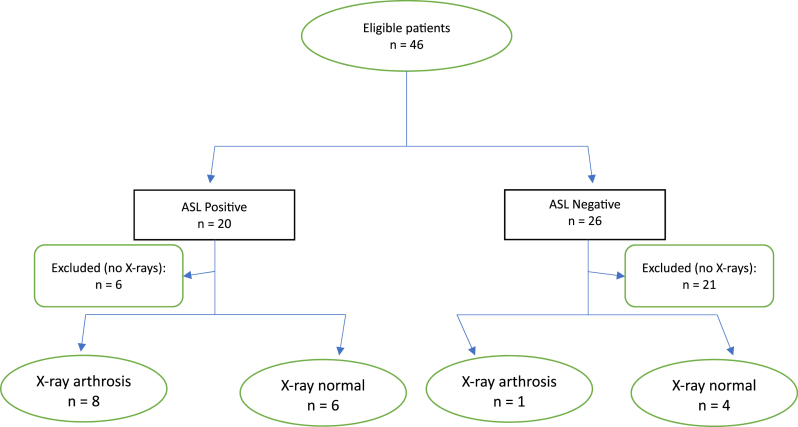
Flow diagram. ASL = Age, Superior cervical/suboccipital pain, exacerbated by Lateral rotation.

For the secondary analysis, in determining if the ASL criteria correlated with C1-2 joint arthrosis on x-ray, defined as severe joint space loss and/or sclerosis, 14 cases and 5 controls were included in the analysis (6 cases and 21 controls were excluded due to the absence of x-rays with sufficient visualization of the lateral atlantoaxial joints) (Fig. 4). Eight cases were positive for C1-2 joint arthrosis on x-ray while 6 were negative, and 1 control was positive while 4 were negative (Table 3). The sensitivity of the ASL criteria was 89 % [95 % CI: 69–100 %], the specificity was 40 % [95 % CI: 10–70 %], and the positive likelihood ratio was 1.5. The PPV and NPV based on a prevalence of 47 % were 57 % [95 % CI: 31–83 %] and 80 % [95 % CI: 45–100 %], respectively. The diagnostic confidence odds were 1.3, with a diagnostic confidence of 0.57.

**Table 3:** Contingency table for Positive ASL criteria and C1-2 joint arthrosis on X-ray.

	X-ray positive joint	X-ray negative joint
Cases (Positive ASL criteria)	8	6
Controls (Negative ASL criteria)	1	4

ASL = Age, Superior cervical/suboccipital, Lateral rotation.

## 5 Discussion

Our pilot study shows that a neck pain patient's history, consisting of the 3 ASL features (Age >65, Superior cervical/suboccipital pain, and exacerbation of pain exclusively or predominantly with cervical spine Lateral rotation to either or both sides), is an acceptable method for diagnosing C1-2 joint edema, with sensitivity and specificity of approximately 80 %. While the cervical facet joints from C2-3 through C6-7 can only be adequately diagnosed through diagnostic medial branch blocks [15], our study shows that clinical evaluation – specifically history – may be useful in the diagnosis of C1-2 joint pain. We suspect that this is true because of the unique anatomy and function of the C1-2 joint compared to the cervical spine facet joints. Since no gold standard diagnostic or clinical test exists for the diagnosis of C1-2 joint pain, we cannot be certain that the presence of edema in our patients indicates that this was in fact the source of their pain. However, while edema of the posterior elements as demonstrated by STIR MRI can be present in asymptomatic individuals, in the lumbar spine, it is statistically more common in those with symptoms [16]. This, in addition to the fact that 13/14 of our cases had edema on the side of the patient's superior cervical pain (11/14 had edema unilaterally, while 2/14 had edema bilaterally), and none of the cases had edema only on the asymptomatic side (the 14th case had edema only in the dens), is highly suggestive that the presence of edema on these patients' MRIs was not an incidental finding.

We suspect that our ASL features may even be a stronger clinical tool than our study showed. Five of the 19 patients with positive ASL criteria had an MRI negative for edema. Of these five patients, one had severe asymmetric arthrosis of the C1-2 joint on MRI, however there was not edema on the STIR images, so the MRI was graded as negative, despite our suspicion that this patient did in fact have C1-2 joint pain. The other four patients had somewhat diffuse pain with somewhat questionable exacerbating factors, however they did strictly meet our predefined ASL criteria, so they were counted as cases in our study (one also had exacerbation of his pain with eye movements). We suspect that a prospective study with more precise focus on the three clinical features would have even greater specificity and positive predictive value. Sensitivity would likely remain similar as MRI is well-known to show anatomic abnormalities in patients without symptoms.

Our study provides support for the findings from the study by Aprill that showed that clinical features may be useful in diagnosing lateral C1-2 joint pain. However, that study investigated a different population of patients, with a median age of 53 and a large majority of patients presenting after an injury, most often a motor vehicle accident. Only one of their patients was reported to have joint arthropathy [9]. Our study was limited to an older population who were suspected of having degenerative C1-2 joint arthrosis based on the ASL criteria.

Our study has limitations. Most importantly, there is no universally accepted gold standard diagnostic or clinical test for pain emanating from the lateral atlantoaxial joints. The C1-2 joints have been shown to have both dorsal [17] and ventral innervation [18], and a reliable and valid diagnostic block paradigm has not been established. While MRI with fat suppression appears promising in the evaluation of a painful spinal segment [12,13], imaging cannot be relied upon as a definitive diagnosis due to the large false positive rates in asymptomatic individuals, especially in the cervical spine [19]. In our study, we showed a 3/22 (14 %) false positive rate of the MRI. However, in our cases, the large majority (11/14) had edema only on the symptomatic side (or on the side of more severe symptoms in patients who had bilateral symptoms), two had edema bilaterally (including the symptomatic side), and one had edema only in the dens. None had edema only on the asymptomatic side, which is further suggestive that the presence of edema in these cases was clinically relevant to their symptoms. Another limitation is the small sample size in our study. However, despite this small sample size, we achieved fairly impressive sensitivity and specificity results, around 80 %, with reasonably small confidence intervals. Additionally, all retrospective analyses contain biases. We did evaluate consecutive patients, which can limit these biases. Another limitation is that all clinical evaluations in this pilot study were performed by one physician, so the generalizability of these findings to other practices is unknown. Given the encouraging results from this study, we plan to address these limitations with a larger, prospective study using multiple providers.

## 6 Conclusion

Three features of the medical history, consisting of age >65, location of pain at the superior cervical/suboccipital region, and exacerbation of pain exclusively or predominantly by lateral cervical spine rotation, referred to as the ASL criteria (Age, Superior, Lateral rotation), have sensitivity and specificity of approximately 80 % in the diagnosis of C1-2 joint edema, which may have clinical implications.

This research did not receive any specific grant from funding agencies in the public, commercial, or not-for-profit sectors.

## Declaration of competing interest

The authors declare the following financial interests/personal relationships which may be considered as potential competing interests:

Joshua Levin reports a relationship with State Farm Insurance Companies that includes: consulting or advisory. Joshua Levin reports a relationship with Liberty Mutual Group Inc that includes: consulting or advisory. Joshua Levin reports a relationship with Allstate Insurance that includes: consulting or advisory. Joshua Levin reports a relationship with Hanover Insurance Group that includes: consulting or advisory. Joshua Levin reports a relationship with GEICO that includes: consulting or advisory. If there are other authors, they declare that they have no known competing financial interests or personal relationships that could have appeared to influence the work reported in this paper.
